# Analysis of Intron Sequence Features Associated with Transcriptional Regulation in Human Genes

**DOI:** 10.1371/journal.pone.0046784

**Published:** 2012-10-17

**Authors:** Huimin Li, Dan Chen, Jing Zhang

**Affiliations:** 1 Laboratory for Conservation and Utilization of Bio-resources, Yunnan University, Kunming, China; 2 School of Mathematics and Statistics, Yunnan University, Kunming, China; 3 School of Mathematics and Computer Science, Yunnan University of Nationalities, Kunming, China; University of Maryland School of Medicine, United States of America

## Abstract

Although some preliminary work has revealed the potential transcriptional regulatory function of the introns in eukaryotes, additional evidences are needed to support this conjecture. In this study, we perform systemic analyses of the sequence characteristics of human introns. The results show that the first introns are generally longer and C, G and their dinucleotide compositions are over-represented relative to other introns, which are consistent with the previous findings. In addition, some new phenomena concerned with transcriptional regulation are found: i) the first introns are enriched in CpG islands; and ii) the percentages of the first introns containing TATA, CAAT and GC boxes are relatively higher than other position introns. The similar features of introns are observed in tissue-specific genes. The results further support that the first introns of human genes are likely to be involved in transcriptional regulation, and give an insight into the transcriptional regulatory regions of genes.

## Introduction

Protein coding genes in eukaryotes contain multiple introns that are cut off in RNA splicing process [1]. The introns have been considered as “junk DNA” for a long time. However, a growing number of recent researches indicate that these so-called “junk DNA” may have important biological functions [2]–[12]. In particular, some studies have suggested that the first introns may play a vital role in transcriptional regulation of genes [13]–[16].

The characteristics of human introns have attracted considerable attention [17]–[22]. The previous work have studied the length and nucleotide content of the introns and found that the first introns are generally longer than other introns and are enriched in C+G and CG dinucleotide compared to other introns [17], [20], [21], suggesting that the longer first introns may be favorable for the presence of regulatory elements [20], [21]. Therefore, it is necessary to study intron length and nucleotide content for evaluating the function of introns in transcription.

The CpG island is also known to be involved in transcriptional regulation of genes. Generally, unmethylated CpG islands are the binding sites of some important transcription factors (TFs), such as Sp1 [23]; DNA methylation is an efficient way to repress gene transcription. Methylation of CpG island can affect gene transcription in two ways: modifying chromatin structure to inhibit the binding of TFs to the promoter regions and alternating binding sites of TFs [24], [25]. Despite many studies focusing on the CpG islands in human genome [26], [27], the special analysis about distributions of these CpG islands in the introns remain poorly understood as far as we know.

Transcriptional regulatory elements in many genes consist of several essential motifs [29]. The most basic and key motifs, including TATA box, CAAT box and GC box, are well known to be bound by general TFs. For example, TATA box is normally bound by the TF TBP in the process of transcription [30]. Therefore, frequent occurrence of TATA, CAAT and GC boxes in sequences is conductive to the bindings of a lot of TFs. In addition, it is interesting to speculate that the frequency of the motif-containing introns might represent the potential of introns in transcriptional regulation. However, to our knowledge, the systematic analysis of these motifs in the introns has not been reported yet.

In this paper, we focus on investigating the intron features associated with transcriptional regulation in human genes. In order to systematically obtain the rational results, we analyze intron features not only in the entire sample genes, but also in introns of tissue-specific (TSP) genes, additionally. Firstly, we compare the intron length in various ways and find that the first introns are generally longer than other introns, consistent with the previous studies [17], [20]. Secondly, the contents of the nucleotides and dinucleotides in the introns are counted and compared. The results indicate that C, G and their dinucleotide compositions are over-represented in the first introns, whereas A, T and their dinucleotide compositions are under-represented. Thirdly, the frequencies of introns containing CpG islands are calculated. The proportion of the CpG-containing first introns is higher than that of other introns. In particular, compared to other position-specific introns, quite a number of the first introns contain CpG islands. In addition, the distributions of the repetitive element are different between the first and non-first introns. Finally, we find that the percentages of the first introns containing TATA, CAAT and GC boxes are relatively higher. These findings further support the hypothesis that the first introns of most human genes may play an important role in transcriptional regulation.

## Materials and Methods

### Sequences of genes

A C++ script was written to extract data from NCBI Genbank database (builder 36.2). Only genes with transcript and coding sequence (CDS) features were utilized. Intron lengths were inferred according to the mRNA location information. Since some genes have multiple transcripts (e.g., genes with alternative splicing), we only retained one transcript feature to avoid duplicate identification of introns for the identical genes. Thus a set of about 185,000 introns were obtained from 19770 intron-containing genes. In order to compare the intron features better, sampled introns were classified by two ways. The first classified pattern is ‘first introns’ which contain all the first introns and ‘non-first introns’ in which all other introns are classified into the same group. The second way is to classify introns based on their position specificity in genes (i.e., the first intron, the second intron, etc.).

We retrieved the TSP genes from the HugeIndex database [31], in which the mRNA expression levels of thousands of human genes can be used for identification of the TSP genes. 19 groups of TSP genes were obtained using the search tool in HugeIndex. Moreover, the housekeeping (HK) genes were obtained from the housekeeping subset of the database. The corresponding introns of the TSP and HK genes were also extracted from the NCBI. The numbers and the names of all groups of genes are listed in Table 1 and Table S1, respectively.

**Table 1:** Comparison of intron lengths

Gene^d^	N(genes)^f^	Aver1^g^	Aver-1^h^	L<exp(L)^i^	L = exp(L)^j^	L>exp(L)^k^	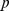 value^l^
All^ e^	19770	13732	4861	39.13	0.02	60.84	∼0
Housekeeping genes (HK)	414	5851	2136	36.75	0	63.25	3.6e–16
Blood (BD)	145	12340	4121	31.39	0	68.61	4.15e–9
Brain (BRA)	260	22986	7814	35.95	0	64.05	1.82e–12
Breast (BRE)	54	15447	12135	52.08	0	47.92	0.9942
Cervix (CX)	53	21039	5109	38.46	0	61.54	1.92e–6
Colon (CO)	37	22325	5605	51.43	0	48.57	3e–3
Endometrium (ENDO)	125	19587	5629	30.83	0	69.17	1.03e–11
Esophagus (ES)	111	15358	4290	33.96	0	66.04	2.96e–5
Kidney (KI)	550	17207	5760	36.55	0	63.45	3.9e–34
Liver (LI)	570	14167	4627	34.39	0	65.61	6.17e–35
Lung (LU)	610	15782	5030	33.56	0	66.44	4.91e–42
Muscle (MU)	513	15910	5269	36.36	0	63.64	1.29e–31
Myometrium (MY)	102	21346	5802	34.65	0	65.35	1.07e–7
Ovary (OV)	228	16622	4483	36.87	0	63.13	4.73e–20
Placenta (PL)	105	18213	4302	37.76	0	62.24	7.87e–8
Prostate (PR)	624	16970	5504	35.85	0	64.15	8.2e–41
Stomach (ST)	31	36466	7048	24	0	76	5.37e–5
Spleen (SP)	24	13525	3173	27.27	0	72.73	1.21e–2
Testes (TE)	25	32527	8853	22.73	0	77.27	3.49e–1
Vulva (VU)	162	15179	3621	33.11	0	66.89	1.34e–11

d: the name of each group ;

e: all sample genes;

f: the number of genes in each group;

g: the average lengths (unit: bp) of the first introns;

h: the average lengths of the non-first introns;

i: the gene proportions observed with the first intron length shorter than their expected length;

j: the gene proportions observed with the first intron length equal to their expected length;

k: the gene proportions observed with the first intron length longer than their expected length;

l: the 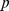 value of KS test.

### Comparisons of intron lengths

The characteristic length (e.g. average length, median) between all sampled first introns and non-first introns were compared. For the genes containing two or more introns, we also compared the length of the first intron with the average intron length (or named as expected length that is defined as the ratio of the overall length of all introns in one gene and the number of introns in this gene) of the same gene. Due to the large sample size and unknown distributions of the intron lengths, two-sample Kolmogorov-Smirnov (KS) tests were used to assess the significance of the length difference between the first introns and non-first introns.

### Analysis of nucleotide content

Nucleotide contents, especially high C+G and CG dinucleotide contents are usually thought to be related to transcriptional regulation of genes [21]. A test pooled variance was used to compare the relative occurrence frequency of certain nucleotide in the first introns and non-first introns [16]. Let 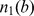
and 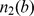 be the occurrences of the nucleotide 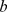 in the first introns and non-first introns, respectively, 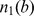
and 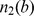
obey binomial distribution. 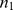 and 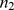 be the total occurrences of all kinds of nucleotide in two sorts of introns, respectively. The occurrence frequency of 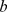 in the first introns and non-first introns are 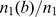 and 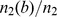, denoted as 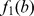 and 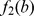, respectively. Then, a null hypothesis is proposed as 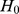: 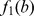 = 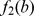 versus 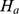: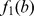 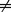 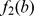. When 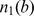 and 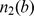 are less than 5, we computed directly the significant level by binomial distribution.

However, for large sample sizes, the exact computation may take too much time. Based on center limit theorem in statistics, the binomial distribution has an asymptotic standard normal distribution as 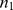 and 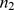 sufficiently large (in general, when 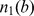 and 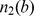 are more than or equal to 5, the sample is considered as larger sample). Since the normal distribution contains real observations, while the binomial distribution only contains integer observations, continuity correction is necessary when using a normal distribution to approximate a binomial distribution. Therefore, the difference in occurrence frequency of 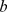 between the first introns and non-first introns is calculated as follows (called 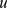-value):



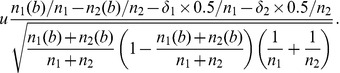
(1)Where


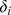
 = 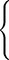 
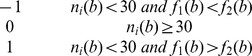
 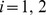.

Namely, 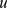
value is performed a continuity correlation when 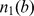 or 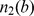 are less than 30.

Choosing significant level 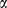 = 0.05, 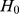 is rejected and 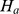 is accepted if 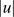
>1.96. This means that the occurrence frequency of 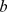 is significantly different between the first introns and the non-first introns. The larger the 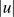 value is, the more significant the difference of occurrence frequency of 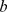 can be seen. In particular, 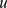
>1.96 means that 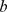 is over-represented in the first introns, while 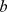 is under-represented in the first introns if 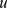
<−1.96.

The similar method and significant level were used to examine the difference in occurrence frequency of dinucleotides between the first introns and non-first introns.

### CpG islands in introns

We searched potential CpG island regions in introns using the method described by Gardiner-Garden and Frommer (1987) [32]. A DNA region can be considered as a CpG island, if (i) it is 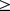
200bp in length; (ii) it has a 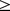
50% G + C content; and (iii) its ratio of observed and expected CG dinucleotide is 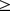 0.6. The expected number of CG dinucleotide in a region is calculated as 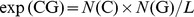
(2)where 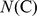 and 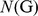 denote the number of nucleotide C and G in the region, respectively; 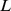 represents the region length. In our analysis, 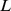 = 200 bp. Note that some introns are shorter than 200 bp, we deemed that these introns contain CpG islands if they satisfy (ii) and (iii).

### TATA, CAAT and GC boxes in introns

The frequencies of introns harboring potential TATA, CAAT and GC boxes were assessed, respectively. The corresponding consensus sequences for the three motifs were TATAWAW (W represents A or T), CCAAT and GGGCGG.

## Results

### Intron lengths

The average intron lengths of the entire samples, HK genes and TSP genes are listed in Table 1. It is interesting that the average length of the first introns is longer than that of the non-first introns. Generally, the average lengths of the first introns are more than twice longer than those of the non-first introns. The most extreme case is observed in stomach-specific genes where the average length of the first introns is more than five times longer than that of non-first introns. The other characteristic lengths of the first introns are also longer than those of the non-first introns. For instance, the median of the first intron length is 3208 bp, whereas, that of the non-first length is only 1446 bp. It is worth noting that the characteristic lengths of both the first introns and non-first introns in HK genes are shorter than those in TSP genes (Figure S1). For example, the average length of the first introns in HK genes is 5851 bp, whereas that in the TSP genes is more than 10000 bp. This indicates that the introns of HK genes tend to be shorter than TSP genes.

**Figure 1:**
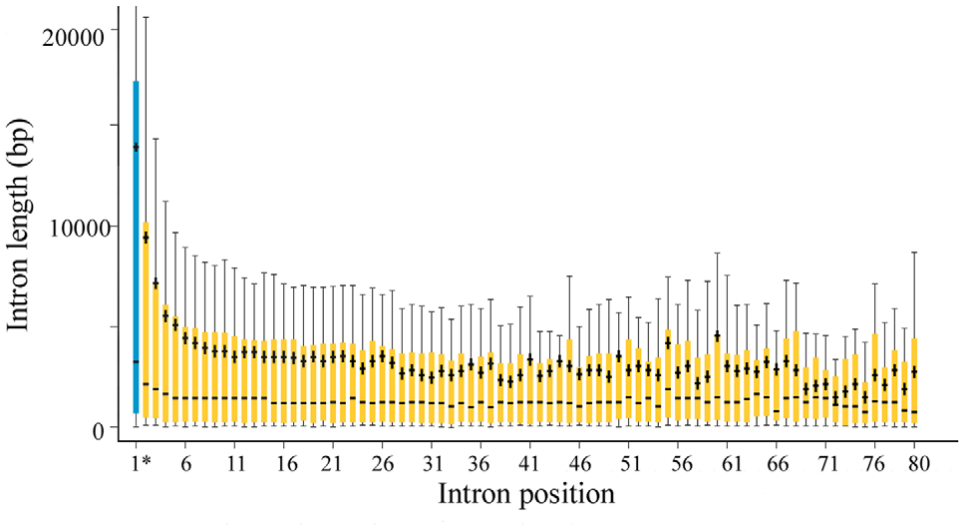
Characteristic lengths of the introns. The bottom and top of the box is the 25^th^ percentile (the lower quartile) and 75^th^ percentile (the upper quartile) of lengths, respectively; the line in the box is the 50^th^ percentile (the median). The lowest and highest datum is the minimum and maximum not considered outliers, respectively, and outliers are not plotted. ‘+’denotes average length of introns with the same position. ‘*’ denotes that the maximum length of the first introns is more than 20000 bp which is not displayed in figure. The situations of introns whose position beyond 80 are not shown since the numbers of sequences are less than 30, hereinafter.

About 2/3 of the first introns are longer than the expected length of the same gene. Except breast and colon-specific genes, the genes with the first introns longer than the expected length are more than the genes with the first introns shorter than the expected length (Table 1). The results suggest that the first introns are longer than the non-first introns in HK genes and almost all TSP genes. In addition, KS tests were performed to compare the lengths of the first introns and non-first introns (Table 1). Significant results were found except for breast and testes-specific genes, suggesting that the length distributions between the first introns and non-first introns are different (KS test: 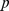
<0.05). We also investigated the characteristic lengths of the position-specific introns to prove the trend that the first introns are longer (Figure 1). We can see that, the average intron lengths, the median lengths, even the lower quartile and the upper quartile lengths of the first introns are obviously longer than those of else other position-specific ones. For example, the lower quartile, the median and the upper quartile length of the first introns is 873 bp, 3208 bp and 11493 bp, respectively, whereas those length of the second introns (that is also significantly longer than introns that followed) is only 669 bp, 2128 bp and 6562 bp. These results further convince the conclusion that the first intron is generally longer in most of the genes, agreeing with and strengthening previous work [20].

### Usages of nucleotides and dinucleotides

**Figure 2:**
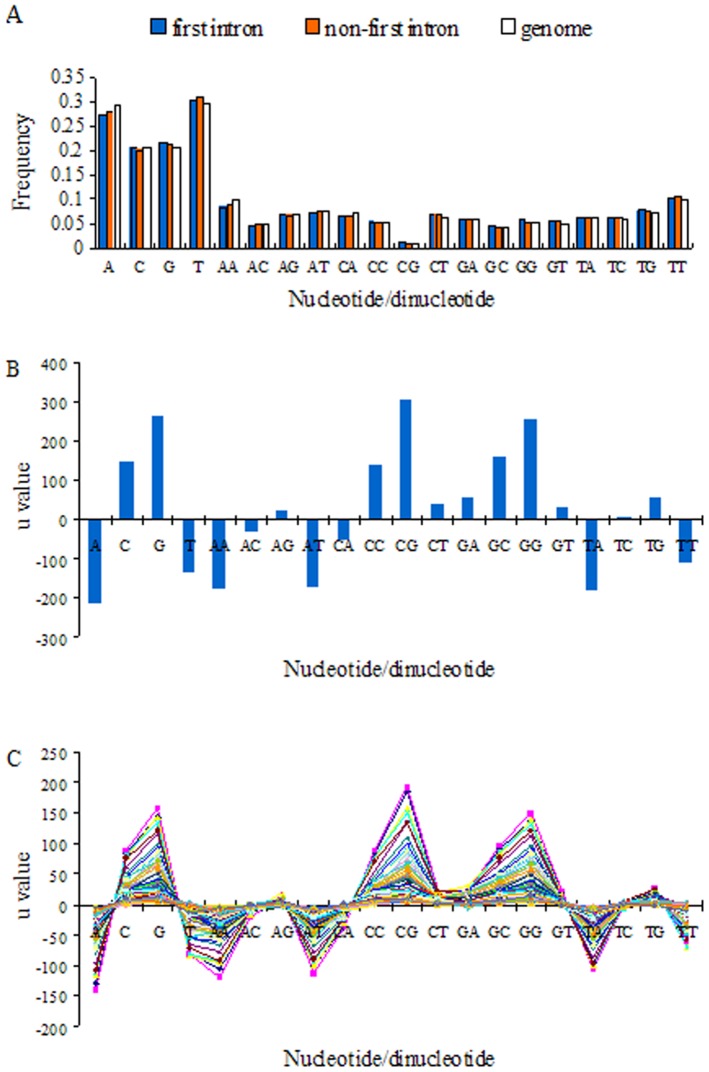
Occurrence frequencies of nucleotides and dinucleotides in introns. (A) Frequencies of nucleotides and dinucleotides in the first introns and non-first introns. (B) u-values of nucleotides and dinucleotides in the first introns relative to the non-first introns. (C) u-values of nucleotides and dinucleotides in the first introns relative to the non-first introns at different positions. One colored polyline represents a group of u-values of all kinds of nucleotides and dinucleotides for the first introns vs. the non-first introns in one specific position. Since we only focus on whether some nucleotides and dinucleotides are over-represented in the first introns, the differences of nucleotide or dinucleotide content between any two position-specific non-first introns do not be concerned.

The contents of nucleotides and dinucleotides in introns are shown in Figure 2. It shows that A+T content is 1.5 times as high as C+G content both in the first introns and in the non-first introns, and the numbers of dinucleotides composed of A and T are obviously larger than those of dinucleotides composed of C and G (Figure 2A). This suggests that higher A+T and their dinucleotide contents are common in introns. However, when 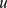-values were used to compare the frequencies of all kinds of nucleotides and dinucleotides between the first introns and non-first introns (Figure 2B), the results were unexpected. The top nucleotides and dinucleotides with the highest 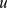-values are C, G and their dinucleotide compositions, whereas the bottom nucleotides and dinucleotides with the lowest 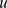-values are A and T and their dinucleotide compositions. The results indicate that the nucleotides or dinucleotides composed of C and G are over-represented in the first introns, whereas nucleotides or dinucleotides composed of A and T are under-represented. It should be noted that among all dinucleotides, CG is the most over-represented (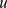 = 305.44, 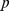 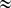
0). We also calculated the nucleotide/dinucleotide frequencies genome-wide and found that the nucleotide/dinucleotide distribution in introns are well match the genome-wide distribution which is AT rich overall (Figure 2A). We found that the CG is still over-represented in the first introns compared the genome (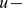
value: C: 41; G: 486.96; CC: 136.08; CG: 336.18; GC: 219.66; GG: 380.10. 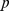
value of them is almost 0). However, this was not completely true when we compared the nucleotide/dinucleotide content differences between the non-first introns and genome (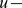
value: C:−184.75; G: 318.33; CC:−19.32; CG: −9.83; GC: 72.37; GG: 154.59). This convinces the higher CG content is general in the first introns.

The 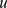-values of nucleotides and dinucleotides were also calculated in HK genes and TSP genes. We found that C, G and their dinucleotide compositions are still over-represented in the first introns of almost all groups. Two exceptions are brain and stomach-specific genes in which C, G, CC and GC are under-represented, while A, T, AA, TA and TT are over-represented. One difference between the two groups of genes is that CG and GG are over-represented in the first introns of brain-specific genes but under-represented in those of stomach-specific genes. Besides, we do not find significant 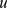-value difference between the first introns vs. the non-first introns in HK genes and those in TSP genes, respectively.

We also carried out a *u* -value analysis to investigate usages bias of nucleotides and dinucleotides in the first introns relative to position-specific non-first introns (Figure 2C). Since we only focus on whether some nucleotides and dinucleotides are over-represented in the first introns, the differences of nucleotide or dinucleotide content between any two groups of position-specific non-first introns do not be concerned. The result shows that compared to most other position-specific non-first introns, C, G and their dinucleotide compositions are over-represented, and A, T and their dinucleotide compositions are under-represented in the first introns. This result suggests that C+G is generally over-represented in the first introns.

### CpG island occurrence

A high content of C+G and CG dinucleotide generally leads to the formation of CpG island, which was found in 40∼50% of human genes in previous studies [26], [27] and 76% of human promoters including the downstream 1000 bases [28]. Since we have found that the majority (95%) of the first exons of human genes are shorter than 1000 bp (data not shown), it could presume that lots of CpG islands located in the first introns. In fact, we found that nearly 75% of the first introns overlap with at least one CpG island, whereas only 30% of the non-first introns overlap with CpG islands. In addition, the fractions of the first introns containing CpG islands in all groups of TSP genes are over 60%, implying that a majority of the first introns contain CpG islands. Another interesting finding is that the first introns of the HK genes have higher CpG island content (85.90%) than those of the TSP genes (<80% except for endometrium and ovary-specific genes), suggesting that the HK genes may have more CpG island-related regulatory elements (Figure S2).

**Figure 3:**
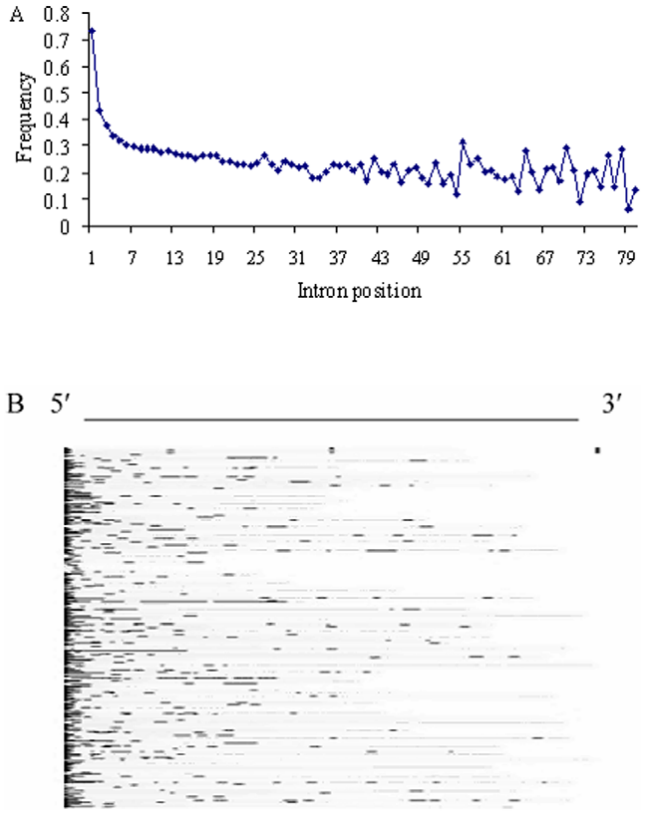
CpG island in the introns. (A) Frequencies of introns containing CpG islands at different positions. (B) Locations of CpG islands in the first introns. 500 introns are selected randomly from the first introns. The CpG islands are represented as black lines in the first introns where other regions are represented as gray spaces. The sequence direction is from 5′-ends to 3′-ends of the first introns.

In order to determine the distribution of CpG islands, we compared the frequencies of the position-specific introns overlapping with CpG islands. The result shows that proportion of the first introns containing CpG islands is far higher than that of any other positional introns (Figure 3A). Interestingly, the proportions of CpG island-containing introns sharply decrease with respect to their intron position in the genes (Figure 3B). For example, less than 45% of the second introns contain CpG islands and 37.6% of the third introns harbor them. Figure 3B displays the CpG islands located in random 500 first introns. The CpG islands appear to cluster near the 5′-end of these introns.

**Figure 4:**
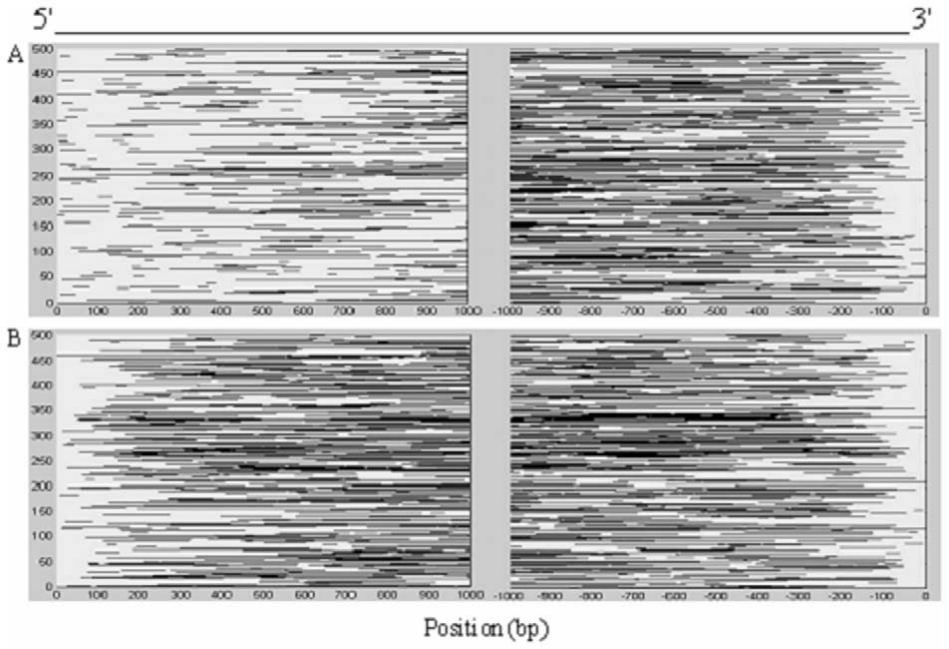
Repetitive elements in the introns. The repetitive elements are represented as black lines in the introns, where other regions are represented as gray spaces. The values along the vertical coordinates represent the numbers of introns (500 introns are selected randomly from the first introns and other introns, respectively). The directions of introns are from 5′-ends to 3′-ends. The nucleotide positions are relative to the splice sites. (A) Repetitive elements in the 5′-ends (left panel) and 3′-ends (right panel) of the first introns. (B) Repetitive elements in the 5′-ends (left panel) and 3′-ends (right panel) of the other introns position. ‘0’ at 5′-end and 3′-end denotes the position of 5′-splice site and 3′-splice site of the introns, respectively.

Repetitive elements are ubiquitous in the mammalian genomes [33], [34] and seldom contain transcriptional regulatory motifs. Therefore, it is expected to observe that there are few repetitive elements in the regions clustered with CpG islands. For this purpose, we analyzed the distribution of interspersed and simple repeats (including SINEs, LINEs, LTR elements, Low-complexity DNA and simple repeats) in the first introns and non-first introns using RepeatMasker software. We found that average 1/3 of one intron, whether the first intron or the non-first intron, contain repetitive elements. However, the distributions of the repetitive elements in these two types of introns are different, especially in the proximity to the splice sites. Figure 4 shows the distributions of repetitive elements in some long human introns (>2000 bp). In general, the distribution of the repetitive elements is asymmetrical between the 5′-end and 3′-end (200 nucleotides away from the splice junctions) of the first introns. Figure 4A demonstrates that there are significantly less repetitive elements at the 5′-ends of the first introns than at the 3′-ends (8.04% near 5′-ends vs. 11.33% near 3′-ends). One sees the same general trends using the (non-parametric) sign-test for differences in the repetitive elements counts in the 5′-end and the 3′-end of the first introns (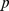
value of sign-test is 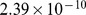). In particular, few repetitive elements are found in the first 1–150 nucleotide positions of the first introns. However, the distribution of repetitive elements in the non-first introns is relatively symmetrical at both the 5′- and 3′-ends of the non-first introns (Figure 4B, 12.13% near 5′-ends vs. 11.34% near 3′-ends 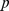
value of sign-test is 0.074). This suggests that the 5′-ends of the first introns may more likely be functional. In view of the different trends of the 5′-ends of the first introns and the non-first introns, it is unlikely that these regions are responsible for splicing regulation. However, in conjunction with the enrichment of CpG islands in this region, the results hint that the 5′-end of the first introns may play an important role in transcriptional regulation of genes.

### Frequency analyses of TATA, CAAT and GC boxes

In order to inspect whether some important transcription factor binding sites (TFBSs)are located in the introns, we calculated the frequencies of introns that contain TATA, CAAT and GC boxes. The results show that, of all the first introns, 56.4% contain TATA boxes; 66.2% and 56.2% contain CAAT and GC boxes, respectively. However the percentages of the non-first introns that contain TATA, CAAT and GC boxes are 49.9%, 52.6% and 20.6%, respectively. This indicates that the three TFBSs occur more frequently in the first introns than in the non-first introns. In particular, the occurrence frequency of the GC box in the first introns is nearly 36% higher than that in the non-first introns, in accordance with previous finding that the first introns contain a larger degree of GC dinucleotide and CpG island than the non-first introns. The frequencies of TATA and CAAT boxes in the first introns are only 6.5% and 13.6% higher than those in the non-first introns. Nevertheless, since nucleotides A and T and their dinucleotide compositions and the CA dinucleotide are under-represented in the first introns, it is reasonable to think that the contents of TATA and CAAT boxes in the first introns are richer than those in the non-first introns.

Similar results are also found in introns of the TSP genes (Figure S3). In all 19 groups of TSP genes, the fractions of the first introns containing above the three TFBSs are higher than those of the non-first introns, while there still are some differences between these groups. In the HK genes, 41% and 53.4% of the first introns have TATA and CAAT boxes, respectively; whereas in most of the TSP genes about 60% and 70% of the first introns have the two boxes. However, it is different for the GC box: the first introns of the HK genes are enriched in GC boxes compared to those of the TSP genes with only a few exceptions. The results suggest that the first introns in the HK genes prefer CG-rich sites while those in TSP genes prefer TATA and CAAT boxes.

**Figure 5:**
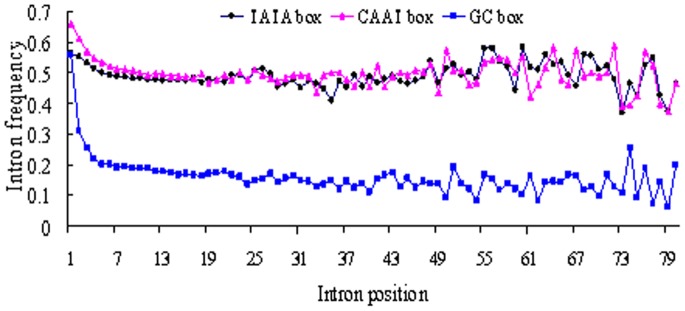
TATA, CAAT and GC boxes frequencies in introns

Our study also reveals that there are differences in frequencies of TATA, CAAT and GC boxes with respect to the intron position (Figure 5). The notable one is that >55% of the first introns contain the GC box, while <30% of the position-specific non-first introns contain the GC box. The proportions of the position-specific introns containing CAAT boxes seem to be little different in the TSP genes, but they are slightly larger in the first introns. The percentage of the first introns containing TATA box is also higher than that of the non-first introns with only several exceptions. Even after masking the repetitive elements in the introns, the frequencies of these two boxes show little differences (data not shown). The results demonstrate that the percentages of the first introns with TATA, CAAT and GC boxes are relatively higher.

### Analysis of introns using alternative introns

It is noted that, we determined the first intron of a gene only based on one transcript of the gene. However, most genes have alternative transcription start sites which maybe affect what is annotated as the ‘first’ intron and the characteristics of the first introns. To counter these problems, we random chose two groups of alternative transcripts from sampled genes and performed the similar analysis for the features of introns. Excitingly, the scores of intron features have no significant difference between two collections of transcripts (data not shown). For instance, the 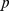 value of KS test for the first intron lengths of two groups of transcripts is 0.55; the average frequencies of CpG-containing the first introns are 79% and 75%, respectively; the frequencies of TATA box, CAAT box and GC box-containing the first introns are 62% versus 64%, 73% versus 71% and 65% versus 62%, respectively, for our sampled transcripts and the random alternative transcripts. The values of intron features even are almost identical in two collections of transcripts. Therefore, it is credible that the effects of selection of transcripts are slight for the features of introns.

## Discussion

Using the introns derived from nearly 20000 intron-containing genes, we analyze the sequence features associated with transcriptional regulation. The various characteristics of the first introns within these genes suggest that the first introns are most likely to be involved in transcriptional regulation. Consistent with previous observations [17], [20], [21], our statistical analysis also reveals that the first introns are longer than the non-first introns. A hypothetical explanation is that the long introns could harbor more functional elements, including those related to transcriptional regulation [20]. The transcription of eukaryotic genes requires interactions between the transcription factors and the cis-acting elements located on DNA. It is not unreasonable to believe that only the long enough introns are capable of accommodating the transcription factor binding sites, and therefore play a role in transcriptional regulation.

Another feature is that C, G and their dinucleotide compositions are over-represented in the first introns, whereas A, T and their dinucleotides are under-represented. In addition, nearly 70% of the first introns contain the CpG island, not only higher than the other introns but also higher than the estimated percentage in entire human genes (40∼50%) [26], [27]. It has been shown that the DNA regions enriched in C+G, CG dinucleotide and CpG island are often related to transcriptional regulation [21]. Our observation may reflect an important role of the first introns in transcriptional regulation. On the one hand, the high contents of C+G, CG dinucleotides and unmethylated CpG islands could provide potential binding sites for some important transcription factors such as Sp1; on the other hand, it offers the chances for transcriptional regulation by CpG methylation. In addition, although the interspersed and simple repeats are widespread in the introns, they are less distributed in the 5′-end of the first introns, suggesting a functional role of the 5′-end of the first introns. In combination with the enrichment of the CpG islands in the 5′-end of the first introns, we propose that this region may take part in transcriptional regulation.

Obvious differences in the distributions of TATA, CAAT and GC boxes between the first introns and the non-first introns are also observed. Generally, the frequencies of these three TFBSs are higher in the first introns than in the non-first introns, suggesting the potential for the first introns to bind TFs and participate in gene expression regulation.

One additional finding of this study is the differences in sequence features between introns within HK genes and TSP genes. The HK genes have shorter introns than the TSP genes. In addition, the frequencies of CpG islands and GC boxes are higher in the HK genes than those in the TSP genes, whereas the HK genes contain fewer TATA and CAAT boxes than the TSP genes. The results hint that there are the different mechanisms of transcriptional regulation between the HK and TSP genes. For example, one possibility is that there are more CG-rich regulatory elements in the HK genes but more AT-rich elements in the TSP genes since the differences of contents of CpG island and TATA box between introns of HK and TSP genes. Besides, there are also differences among the introns within different kinds of TSP genes, indicating the laws of transcriptional regulation between these groups of TSP genes are not the same. Since the differences between HK genes and TSP genes, our next plan is to detect the transcriptional regulatory elements located in the first introns of HK genes and each group of TSP genes and explore the transcriptional regulation mechanism concerned with introns in these genes.

## Conclusion

Our results support the hypothesis that the first introns may contain transcriptional regulatory elements and provide intriguing hints into the regulatory potential of the first introns. Therefore, the promoter regions might be extended to at least the first introns while studying the transcriptional regulations of genes.

## Supporting Information

Table S1 List of houskeeping and tissue-specific genes. (XLS)
(XLS)

Figure S1 Characteristic lengths of introns in housekeeping and tissue-specific genes. (TIF)
(TIF)

Figure S2 Frequencies of introns overlapped with at least one CpG island in housekeeping and tissue-specific genes. (TIF)
(TIF)

Figure S3 TATA, CAAT and GC boxes frequencies of introns in housekeeping and tissue-specific genes. (A) Frequency of introns with TATA boxes. (B) Frequency of introns with CAAT boxes. (C) Frequency of introns with GC boxes. (TIF)
(TIF)
