# Supplementary figures and images for: Analysis of Intron Sequence Features Associated with Transcriptional Regulation in Human Genes
Source: PLoS One. 2012 Oct 17;7(10):e46784. doi: 10.1371/journal.pone.0046784 (PMC3474797; doi:10.1371/journal.pone.0046784)

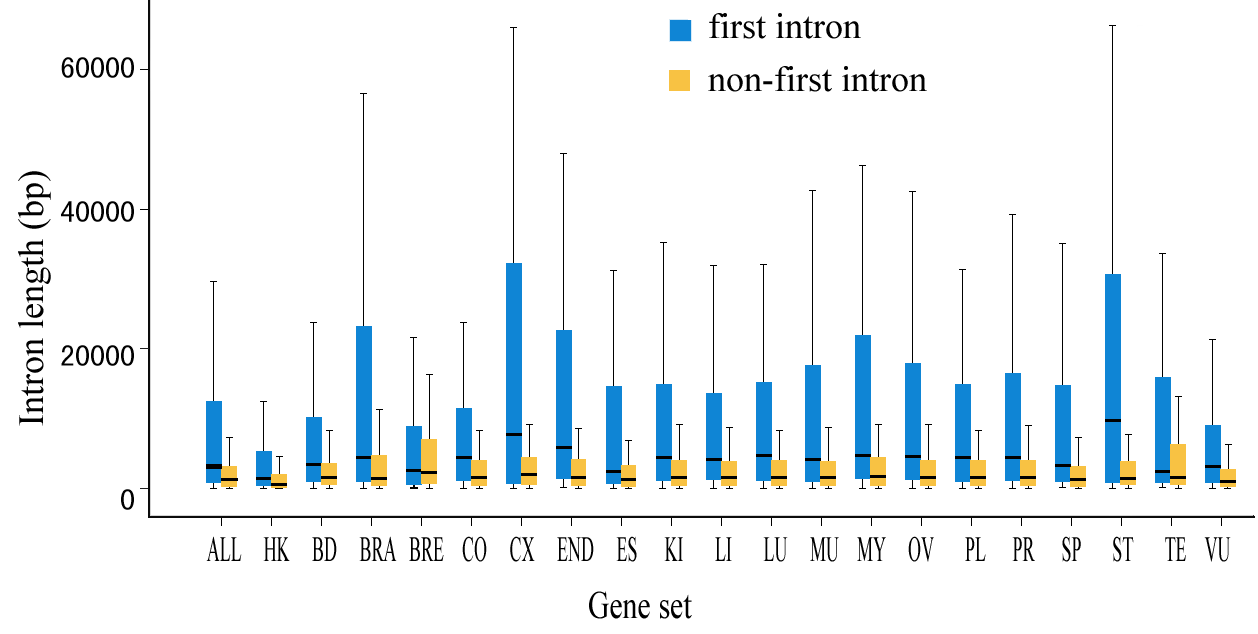

Supplement: Figure S1 — Characteristic lengths of introns in housekeeping and tissue-specific genes. (TIF) (TIF) [file pone.0046784.s002.tif]

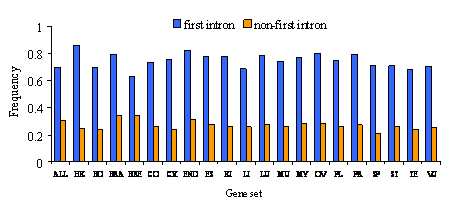

Supplement: Figure S2 — Frequencies of introns overlapped with at least one CpG island in housekeeping and tissue-specific genes. (TIF) (TIF) [file pone.0046784.s003.tif]

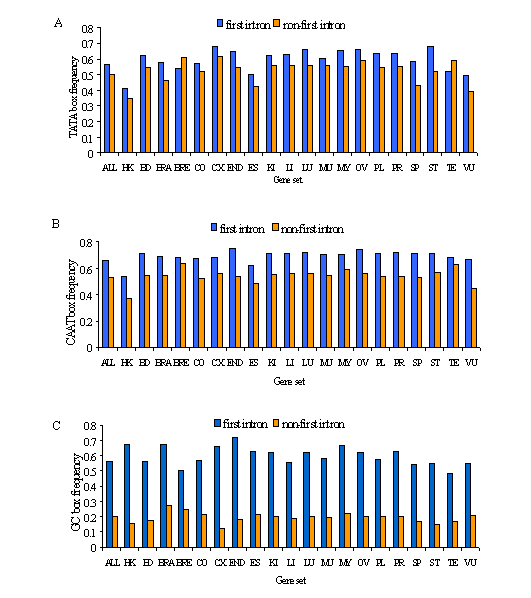

Supplement: Figure S3 — TATA, CAAT and GC boxes frequencies of introns in housekeeping and tissue-specific genes. (A) Frequency of introns with TATA boxes. (B) Frequency of introns with CAAT boxes. (C) Frequency of introns with GC boxes. (TIF) (TIF) [file pone.0046784.s004.tif]
